# Coffee vs. Gout

**Published:** 1875-06

**Authors:** 


COFFEE VS. GOUT.—Persons troubled with rheumatism or gout are recommended to put a tablespoonful of green or unroasted coffee in half a tumbler of pure water, of the temperature of the surrounding air, and after it has stood some twenty-four hours to drink off the liquid immediately upon getting up in the morning. The glass is again to be filled with water and the drinking practiced as before. The liquid obtained in this way is of a green color, more or less tinged with blue, according to the kind of coffee used. Without undertaking to determine the chemical composition of the water, it is said that the grains swell considerably and sometimes sprout, throwing off small bubbles of gas, which we presume to be carbonic acid, and we incline to think that the liquid attacks rather the effects of the malady than the disease itself, suppressing the former from day to day, while the latter remains, thus rending a daily use of the remedy necessary.

